# Renewed appreciation of double atrial septum: a rare but important anomaly in clinical practice

**DOI:** 10.1093/ehjcr/ytaf121

**Published:** 2025-03-07

**Authors:** Mengna Zhang, Zhiyong Shi, Xiaohan Yang, Xinbo Zhong

**Affiliations:** Department of Echocardiography, Fuwai Shenzhen Hospital, Chinese Academy of Medical Sciences, No. 12 Langshan Road, Nanshan District, Shenzhen 518020, China; Department of Echocardiography, Fuwai Shenzhen Hospital, Chinese Academy of Medical Sciences, No. 12 Langshan Road, Nanshan District, Shenzhen 518020, China; Department of Cardiovascular Surgery, Fuwai Shenzhen Hospital, Chinese Academy of Medical Sciences, No. 12 Langshan Road, Nanshan District, Shenzhen 518020, China; Department of Echocardiography, Fuwai Shenzhen Hospital, Chinese Academy of Medical Sciences, No. 12 Langshan Road, Nanshan District, Shenzhen 518020, China

## Summary

Double atrial septum is a rare cardiac anomaly with an unclear pathogenesis, which may increase the risk of thrombosis within the interatrial space and systemic emboli. Most reported cases were diagnosed through imaging techniques. The present case, with imaging manifestations similar to those of most reported cases, represents the first comprehensive description of its anatomical and pathological features. Our findings can provide valuable insights into its pathogenesis, classification, differential diagnosis, and management.

## Case description

**Figure 1:**
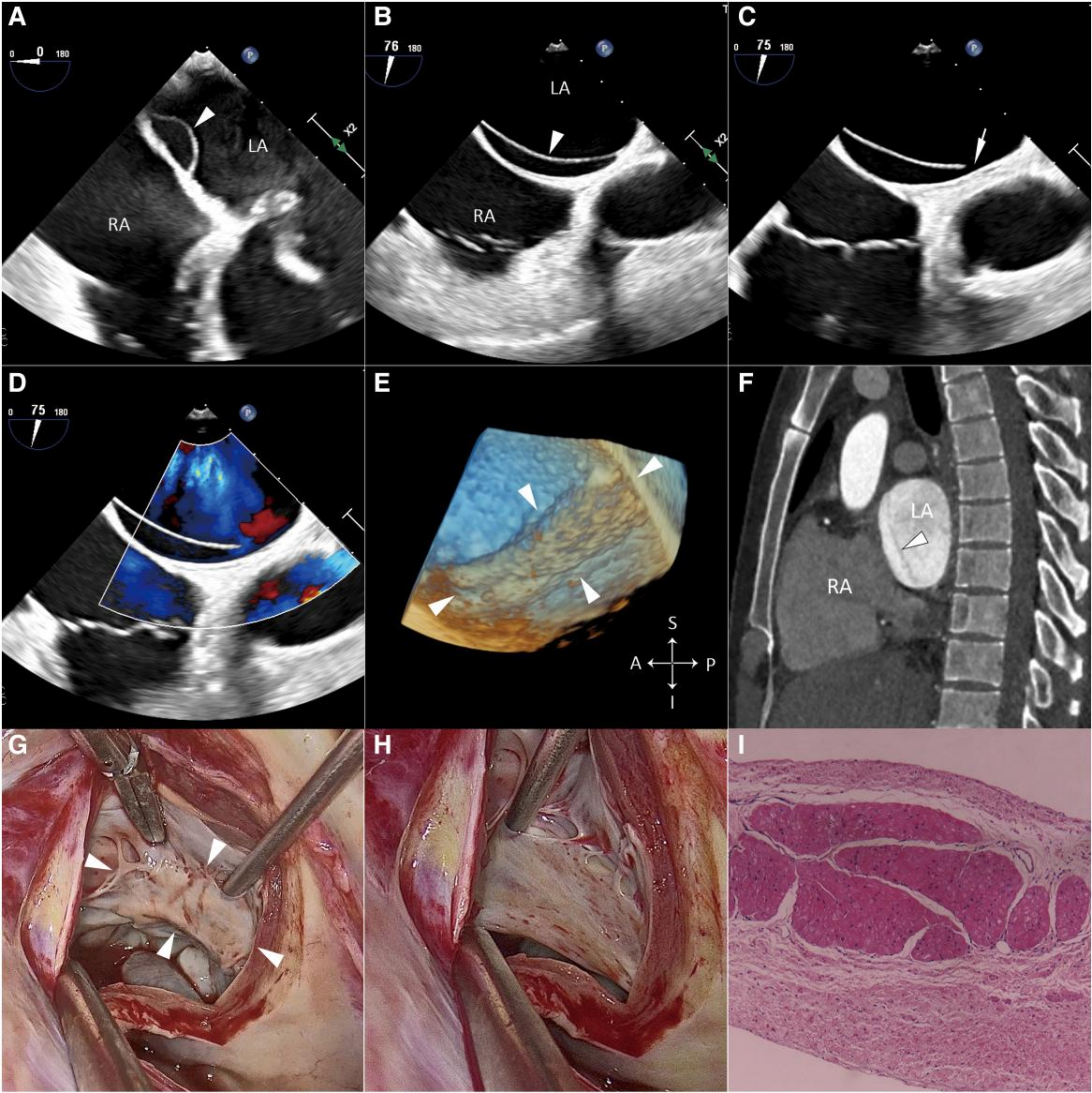
(*A* and *B*) Transesophageal echocardiogram showing a thin membrane (arrowhead) adjacent to the atrial septum, separating the interatrial space from the left atrium. (*C* and *D*) Transesophageal echocardiogram showing fenestration (arrow) on the anterior aspect of the membrane, allowing low-flow communication between the compartments. (*E*) 3D TEE showing the membrane (arrowheads) from the left atrial perspective. (*F*) Contrast-enhanced computed tomography showing the membrane (arrowhead) in the left atrium. (*G* and *H*) Video-assisted thoracoscopic cardiac surgery showing the membrane and multiple fenestrations at its base. (*I*) Histopathological examination of the resected membrane showing an endocardial surface overlying myocardium. TEE, transesophageal echocardiogram.

A 33-year-old male was referred to our centre after a heart murmur was identified during a routine examination. He reported no history of dyspnoea, palpitations, syncope, or chest pain. The results of electrocardiogram were within normal limits. An enlarged left cardiac border was observed in the chest X-ray. Transthoracic echocardiography showed mitral valve prolapse with severe mitral regurgitation. Key measurements included a left ventricular end-systolic diameter of 40 mm, a left atrial anteroposterior diameter of 55 mm, a left ventricular ejection fraction of 61%, and an estimated pulmonary artery systolic pressure of 37 mmHg. No left-sided obstructive lesions were observed. During a transesophageal echocardiogram (TEE), a thin membrane adjacent to the atrial septum was incidentally discovered (*Figure 1A* and *B*, arrowhead). It separated an interatrial space from the left atrium, displaying multiple fenestrations at its anterior (*Figure 1C*, arrow) and posterior (see Supplementary material online, *Figure S1*) aspects, enabling low-flow communication between compartments (*Figure 1D*; Supplementary material online, *Videos S1* and *S2*). The size of the membrane, measured using 3D TEE, was 43 × 15 mm, extending from posterosuperior to anteroinferior direction when viewed from the left atrial perspective (*Figure 1E*). Similar findings were demonstrated by contrast-enhanced computed tomography (*Figure 1F*), with all pulmonary veins drained into the left atrium. Finally, the patient was diagnosed with double atrial septum (DAS) and mitral valve prolapse.

During video-assisted thoracoscopic cardiac surgery, multiple fenestrations at the base of the membrane were observed (*Figure 1G* and *H*), along with an intact atrial septum. Subsequently, the membrane was completely resected (see Supplementary material online, *Figure S2* and *Video S3*) due to concerns about potential thrombus formation. The mitral valve repair was performed successfully. Histopathological examination of the resected membrane revealed an endocardial surface with underlying myocardium (*Figure 1I*). The patient underwent an uneventful post-operative recovery and was discharged 1 week after surgery. At the 1-year follow-up, the patient remained asymptomatic, with an echocardiographic evaluation demonstrating no abnormalities.

The DAS is a rare cardiac anomaly; approximately 40 cases have been reported in the literature. In most reported cases, an accessory fenestrated membrane is found in the left atrium, as observed in our case, which may lead to stagnation in the interatrial space and serving as a nidus for thromboembolism,^1^ similar to that observed in the left atrial appendage. To our knowledge, this is the first comprehensive anatomical and pathological depiction of DAS, providing renewed insights. On 2D imaging, DAS may be misdiagnosed^2^ as a left atrial septal pouch, which is a considerably smaller thrombogenic blind sac^3^ resulting from incomplete fusion of the septum primum and secundum. Moreover, accurate recognition of the 3D morphology of DAS can facilitate transseptal puncture in catheter-based interventions.

## Supplementary Material

ytaf121_Supplementary_Data

## Data Availability

The data underlying this article will be shared upon reasonable request to the corresponding author.

## Notes

**Consent:** The authors confirm that written consent for submission and publication of this case report, including images and associated text, has been obtained from the patient in line with the COPE guidelines.

**Funding:** This work was supported by the Sanming Project of Medicine in Shenzhen (SZSM202011013), Shenzhen Clinical Research Center for Cardiovascular Disease Fund (no. 20220819165348002) and Guangdong Medical Research Foundation (A2022286).
