# Supplementary figures and images for: Renewed appreciation of double atrial septum: a rare but important anomaly in clinical practice
Source: Eur Heart J Case Rep. 2025 Mar 7;9(3):ytaf121. doi: 10.1093/ehjcr/ytaf121 (PMC11940737; doi:10.1093/ehjcr/ytaf121)

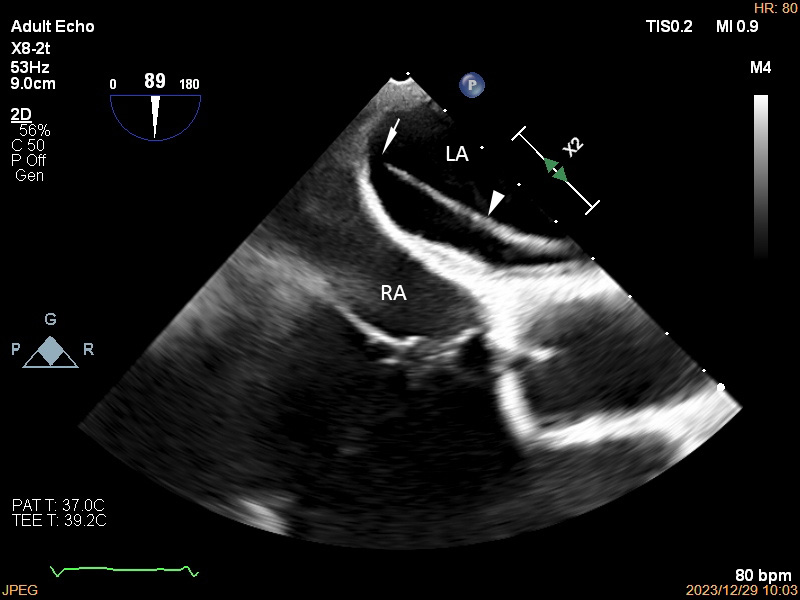

Supplement: ytaf121_Supplementary_Data [file ytaf121_supplementary_data.zip › Figure S1.jpg]

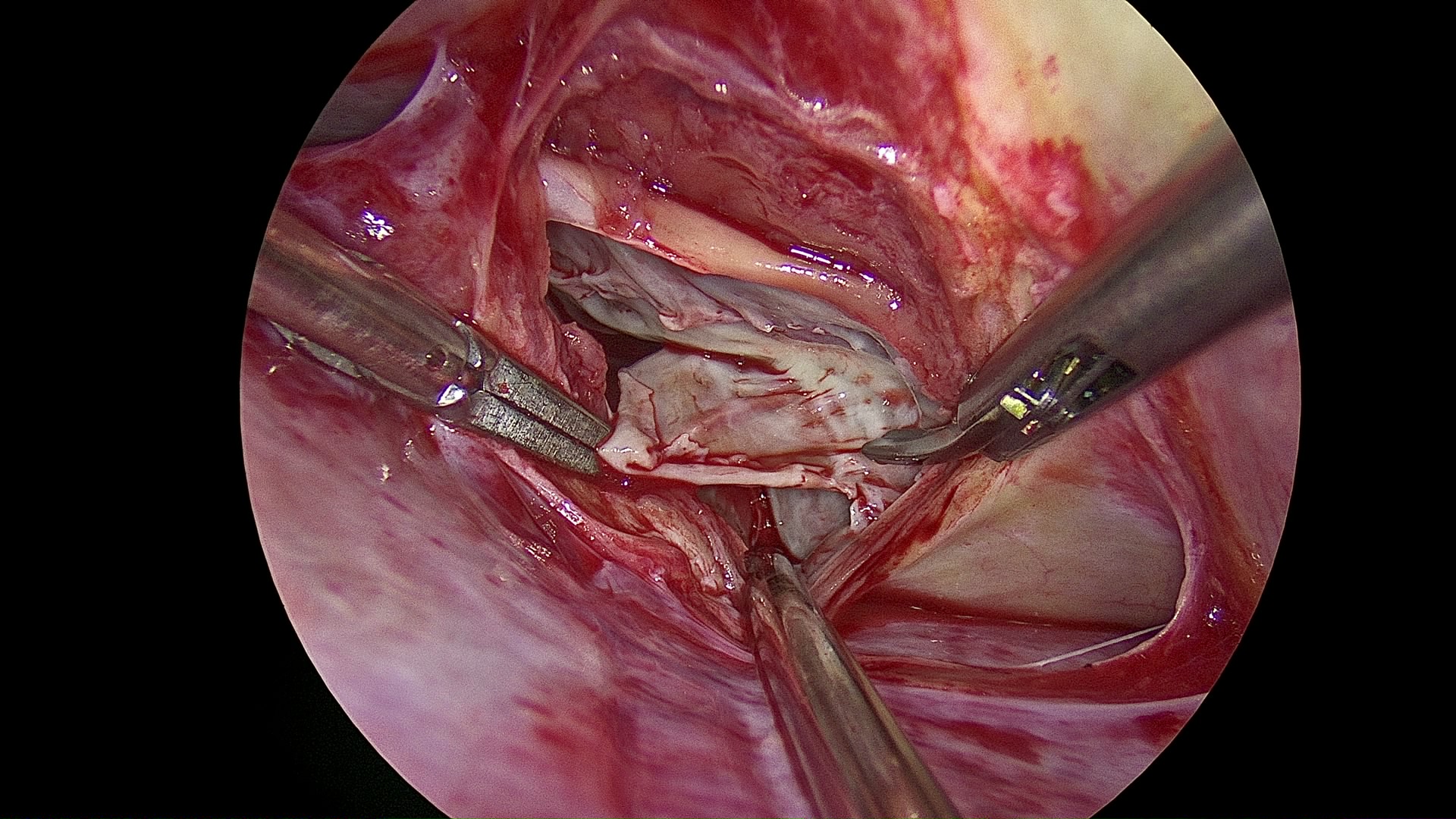

Supplement: ytaf121_Supplementary_Data [file ytaf121_supplementary_data.zip › Figure S2.jpg]
